# Galectin-3 inhibitor selvigaltin dosed therapeutically *in vivo* in a rabbit model of metabolic syndrome induced by high-fat diet: effects on rabbit differentiated adipocytes

**DOI:** 10.3389/fphys.2026.1710592

**Published:** 2026-07-09

**Authors:** Gabriele Acciai, Paolo Comeglio, Sandra Filippi, Ilaria Cellai, Elena Rapizzi, Tommaso Mello, Ian Holyer, Fredrik R. Zetterberg, Andrea Galli, Linda Vignozzi, Robert J. Slack, Mario Maggi

**Affiliations:** 1 Department of Experimental and Clinical Biomedical Sciences “Mario Serio”, University of Florence, Florence, Italy; 2 Department of Neurosciences, Psychology, Drug Research and Child Health (NEUROFARBA), University of Florence, Florence, Italy; 3 Galecto Biotech AB, Copenhagen, Denmark; 4 Interuniversity Consortium “Istituto Nazionale Biostrutture e Biosistemi” (INBB), Rome, Italy

**Keywords:** adipocytes, galectin-3 inhibitor, inflammation, metabolic syndrome, mitochondria, selvigaltin, visceral fat

## Abstract

**Introduction:**

Metabolic syndrome (MetS) is an increasingly prevalent condition characterized by visceral obesity, insulin resistance, dyslipidemia, hypertension, and chronic low-grade inflammation. Impaired preadipocyte differentiation contributes to metabolic disturbances, inflammation, and mitochondrial dysfunction. Galectin-3 (Gal-3) plays a key role in promoting visceral adipose tissue (VAT) inflammation, fibrosis, and mitochondrial impairment. In a preclinical rabbit model of MetS, treatment with the Gal-3 inhibitor selvigaltin reduced VAT mass, indicating potential therapeutic value. This study seeks to clarify how selvigaltin influences the metabolic function of differentiation induction medium (DIM)-induced adipocytes mitochondria, potentially offering a new strategy for treating MetS.

**Methods:**

Male New Zealand White rabbits were housed individually under standard conditions. Following one week on a regular diet (RD), they were randomly assigned to one of the following groups for 12 weeks: RD with vehicle, high-fat diet (HFD) with vehicle, or HFD with selvigaltin (0.3, 1.0, or 5.0 mg/kg), administered orally once daily, five days a week, starting from week 9. Visceral adipose tissue (VAT) was collected, and preadipocytes were isolated and induced to differentiate *in vitro* into DIM-differentiated adipocytes. Subsequent experiments examined mitochondrial dynamics and morphology, superoxide production, oxygen consumption, lipid droplets formation, and target genes mRNA expression.

**Results:**

HFD in rabbits induces features of metabolic syndrome, including increased visceral fat, mitochondrial dysfunction, oxidative stress, and lipid accumulation in visceral adipose tissue. Treatment with Gal-3 inhibitor selvigaltin reversed many of these changes, specifically, improving DIM-differentiated adipocytes mitochondrial morphology and dynamics, reducing oxidative stress and lipid droplet size. Furthermore, the HFD induced upregulation of inflammatory markers (COX2, IL-1β, IL-6, TLR2, TLR4, TNFα) and immune cell polarization transcription factors (GATA3, TBX21), all noticeably decreased by selvigaltin treatment.

**Discussion:**

This study highlights mitochondrial dysfunction as a key factor in VAT impairment. In DIM-differentiated adipocytes, selvigaltin improves mitochondrial structure, dynamics, and function, reduces oxidative stress and lipid droplet size, and lowers the mRNA expression of proinflammatory-related genes. It also promotes a shift toward a healthier, more metabolically active adipocyte phenotype. Selvigaltin restores mitochondrial health, suggesting its potential as a therapeutic option for metabolic disorders involving VAT dysfunction. Further studies are needed to confirm its clinical relevance.

## 1 Introduction

Metabolic syndrome (MetS) is a complex and multifactorial pathological condition characterized by the coexistence of several metabolic abnormalities, including visceral obesity, insulin resistance, dyslipidaemia, hypertension, and low-grade chronic inflammation (Eckel et al., 2005). According to recent epidemiological data, MetS affects between 14% and more than 30% of the global adult population, and its global prevalence is steadily increasing, driven by lifestyle-related factors such as excessive caloric intake, physical inactivity, and aging (Saklayen, 2018; Jamali et al., 2024; Obeidat et al., 2025). MetS is currently recognized as one of the major risk factors for type 2 diabetes, cardiovascular diseases, and metabolic dysfunction–associated steatotic liver disease (MASLD) (Eslam et al., 2020), formerly known as non-alcoholic fatty liver disease (NAFLD) (Byrne and Targher, 2015).

Visceral adipose tissue (VAT) expansion and dysfunction are widely recognized as critical factors in MetS and remain a central topic in current research (Chait and den Hartigh, 2020). Unlike subcutaneous fat, VAT displays a stronger association with systemic metabolic impairment, due to its heightened inflammatory activity and endocrine properties. Indeed, visceral adiposity correlates strongly with insulin resistance and inflammatory markers, emphasizing its central role in the pathogenesis of MetS (Chait and den Hartigh, 2020; An et al., 2023). Therefore, the functional integrity of the adipose tissue, and specifically the ability of visceral preadipocytes to differentiate into metabolically competent adipocytes, plays a pivotal role in modulating the pathological impact of visceral obesity (Vishvanath and Gupta, 2019).

Upon exposure to adipogenic stimuli, isolated visceral preadipocytes can generate mature adipocytes capable of lipid storage, insulin responsiveness, and endocrine signalling. However, under conditions of sustained metabolic stress - such as chronic overnutrition or a high-fat diet - the adipogenic potential of preadipocytes may be severely compromised, as already demonstrated in both humans and animal models (Maneschi et al., 2016; Liu et al., 2017; Comeglio et al., 2018).

This dysfunction leads to the emergence of hypertrophic, metabolic dysfunctional adipocytes and contributes to the perpetuation of local and systemic inflammation and insulin resistance (Maneschi et al., 2016; Bedossa et al., 2017). Several studies have shown that an impaired adipogenic capacity is linked to a pro-inflammatory microenvironment, characterized by elevated levels of cytokines such as TNFα, oxidative stress, and fibrogenic signalling (Yang et al., 2017; Ma et al., 2020; Yu et al., 2024). These conditions inhibit adipocyte differentiation and favour the emergence of fibro-inflammatory phenotypes.

Mitochondrial dysfunction in adipocytes, characterized by impaired dynamics and dysmetabolism, is now recognized as a hallmark of adipose tissue impairment (Woo et al., 2019; Xia et al., 2024). Mitochondrial defects in adipocytes limit energy metabolism, reduce ATP production, and amplify inflammatory signalling, thereby exacerbating systemic insulin resistance (Heinonen et al., 2020).

Among the molecular factors involved in VAT dysfunction, galectin-3 (Gal-3) has emerged as a potential key player (Kiwaki et al., 2007; Baek et al., 2015; Zhu et al., 2022). Circulating Gal-3 levels are increased in obesity and diabetes, showing a correlation with insulin resistance and cardiometabolic risk, suggesting that Gal-3 produced in inflamed VAT may enter the bloodstream and exert systemic-endocrine effects (Weigert et al., 2010; Nayor et al., 2015; Menini et al., 2016). Gal-3 is abundantly expressed in the inflammatory microenvironment of VAT, where it promotes pro-inflammatory macrophage polarization and extracellular matrix deposition (Martínez-Martínez et al., 2016; Lu et al., 2020; Shan et al., 2024). Elevated levels of Gal-3 have been associated with impaired mitochondrial function, inflammatory conditions and fibrosis in adipose tissue.

Beyond adipose tissue, Gal-3 has been implicated in the pathogenesis of cardiovascular remodelling and tissue fibrogenesis through the deposition of collagen (Blanda et al., 2020), further supporting its potential relevance in the multisystem manifestations of MetS. In the heart and vasculature, Gal-3 acts locally by stimulating fibroblast proliferation and activation, amplifying TGF-β/Smad signalling and enhancing collagen deposition. Consequently, genetic or pharmacological inhibition of Gal-3 reduces cardiac fibrosis and improves tissue remodelling (Gehlken et al., 2018; Xiao et al., 2020). Consistent with these mechanisms, higher circulating Gal-3 levels have been associated with heart failure severity, adverse cardiovascular events, and mortality in clinical cohorts (Cheng et al., 2022; Zaborska et al., 2023).

Gal-3 binds cell-surface glycoproteins via its carbohydrate-recognition domain (CRD), and oligomerization forms a multivalent lattice that cross-links surface oligosaccharides, modulating receptor retention, lateral mobility, and extracellular matrix dynamics (Nabi et al., 2015). Genetic deletion and pharmacological inhibition studies, including the CRD-specific inhaled inhibitor TD139, demonstrate that Gal-3 is a critical mediator in multiple cardiac disease models (González et al., 2016; Mosleh et al., 2018; Nguyen et al., 2019). The orally available small-molecule inhibitor selvigaltin (formerly GB1211) selectively targets the Gal-3 CRD with nanomolar affinity, blocking ligand interactions and downstream profibrotic and inflammatory signalling. It was developed through medicinal chemistry optimization of an α-D-galactopyranoside scaffold (Slack et al., 2021; Zetterberg et al., 2022; Aslanis et al., 2023).

In a previous study, we have evaluated the therapeutic effects of the Gal-3 selective inhibitor selvigaltin in a non-genomic preclinical rabbit model of metabolic-associated steatohepatitis (MASH) induced by high-fat diet (HFD) (Comeglio et al., 2024). This animal model faithfully reproduces also the phenotype of human MetS, including hypertension, hyperglycaemia, dyslipidaemia, glucose intolerance, visceral fat accumulation, liver steatosis and fibrosis (Filippi et al., 2009; Morelli et al., 2012; Vignozzi et al., 2014; Maneschi et al., 2016; Comeglio et al., 2018).

VAT accumulation, rather than overall body weight, drives hyperglycemia and hyperlipidemia, while elevated triglycerides contribute to ectopic lipid deposition and tissue inflammation, further exacerbating insulin resistance (Virtue and Vidal-Puig, 2010; Snel et al., 2012).

Treatment with selvigaltin, in addition to ameliorating liver fibrosis, showed an improvement in VAT weight, with a significant reduction observed in selvigaltin-treated animals, compared to those on HFD (Comeglio et al., 2024). These findings have prompted the hypothesis that pharmacological inhibition of Gal-3 could represent a promising therapeutic strategy for improving adipose tissue function in MetS. However, the cellular mechanisms underlying these beneficial effects remain to be fully elucidated. While the role of Gal-3 in tissue inflammation and fibrosis has been explored, its impact on the intrinsic metabolic phenotype of visceral preadipocytes remains to be fully characterized. To our knowledge, no prior studies have addressed how *in vivo* Gal-3 inhibition may influence the functional trajectory of adipocytes in a diet-induced model of metabolic dysfunction.

This study was designed to determine whether *in vivo* Gal-3 inhibition could support the maintenance or restoration of a healthy metabolic phenotype in differentiation induction medium (DIM)-differentiated adipocytes, potentially contributing to the amelioration of VAT dysfunction in the context of MetS.

## 2 Materials and methods

### 2.1 MetS rabbit model experimental plan

In a previous study, our group used a total number of 44 male New Zealand White rabbits (16-week old, Charles River Laboratories, Calco, Italy), weighing about 3kg, to investigate the efficacy of Gal-3 inhibitor selvigaltin in reducing hepatic abnormalities in this animal model of HFD-induced MASH/MetS (Comeglio et al., 2024).

Briefly, after one week of acclimation on regular diet (RD), animals were randomly assigned to either continue on RD (control group) or switch to a high-fat diet (HFD; standard chow supplemented with 0.5% cholesterol and 2.5% vegetable fat) for 12 weeks. For the final four weeks of the feeding period (weeks 9-12), HFD-fed rabbits were subdivided into treatment groups receiving Gal-3 inhibitor selvigaltin by oral gavage at doses of 0.3 (HFD + 0.3mg), 1.0 (HFD + 1.0mg), or 5.0 (HFD + 5.0mg) mg/kg, once daily, 5 days per week. RD and untreated HFD rabbits received the vehicle only (RD+Veh and HFD+Veh groups, respectively). At the end of 12 weeks, rabbits were fasted overnight and euthanized with an intravenous overdose of sodium thiopental (200 mg/kg). At sacrifice, we collected visceral adipose tissue (VAT) was collected and weighed, and biopsies were immediately either processed for preadipocyte isolation (n=6 animals for each experimental group) or snap-frozen/stored at -80 °C for later analyses.

All the experimental procedures were conducted using the facilities of the Department of Biomedical Experimental and Clinical Sciences “Mario Serio” and Department of Experimental and Clinical Medicine, and those of CE.S.A.L. (Centro Stabulazione degli Animali da Laboratorio), and NEUROFARBA Department, University of Florence, Italy.

### 2.2 Ethical statement

All animals received humane care, and animal handling complied with the regulations of Animal Welfare Body of the University of Florence, Florence, Italy, in accordance with the Italian Ministerial Law n. 26/2014. The study was approved by the Ministry of Health authorization n. 146/2021-PR.

Animal experiments conformed to the Animal Research: Reporting of *In Vivo* Experiments (ARRIVE) guidelines (http://www.nc3rs.org.uk/arrive-guidelines).

### 2.3 Metabolic and biochemical analyses

Metabolic profiling and hemodynamic evaluation were conducted in all experimental groups to assess the systemic impact of the HFD and the effects of selvigaltin treatment, as previously described (Comeglio et al., 2024). Measurements were conducted under standardized environmental conditions and with minimal animal handling to reduce stress-induced variability.

### 2.4 Isolation, characterization, and *in vitro* differentiation of rabbit VAT preadipocytes

Preadipocytes (rPADs) were isolated from rabbit VAT samples (n=6 for each experimental group) as previously described (Maneschi et al., 2016; Comeglio et al., 2018). Briefly, fresh *ex-vivo* VAT, after being cut into small fragments with sterile scalpel, was digested in Dulbecco’s PBS containing collagenase (0.1% Type II collagenase; Sigma-Aldrich, St. Louis, MO) in a shaking water bath at 37 °C for 1 h. The digestion was stopped by adding an equal volume of Dulbecco’s Modified Eagle’s Medium (DMEM) supplemented with 10% fetal bovine serum (FBS), and the cell suspension was filtered through a 150 μm nylon mesh to remove debris. The filtrate was centrifuged at 2000x*g* for 10 min at room temperature to pellet the stromal-vascular fraction containing preadipocytes. The pellet was resuspended in erythrocyte lysis buffer (155 mM NH_4_Cl, 10 mM KHCO_3_, 0.1 mM EDTA) for 5 min to eliminate red blood cells, then washed and re-pelleted by centrifugation. The resulting preadipocyte pellet was resuspended in complete growth medium (DMEM high glucose, 10% FBS, 100 U/mL penicillin, 100 μg/mL streptomycin, 2 mM L-glutamine, and 1 μg/mL amphotericin B) and cells were seeded in culture dishes for the amplification. Cultures were maintained at 37 °C in a humidified 95% air/5% CO_2_ atmosphere. After 24–48 h, any non-adherent cells and debris were removed by washing with PBS, and the medium was replaced. rPADs were grown to 80-90% confluence in complete growth medium, which was reached after 4–5 days. Finally, the cells were trypsinized (PBS, 1% trypsin), aliquoted and stored at -80 °C for later use. Preadipocytes were characterized as previously described (Maneschi et al., 2016). For each experimental group, adipogenic differentiation of rPADs was induced using DIM, a standard differentiation induction medium (Student et al., 1980). Upon reaching confluence (designated day 0), the culture medium was switched to DIM, consisting of DMEM supplemented with 5% (v/v) FBS, 0.25 μM dexamethasone, 0.5 mM 3-isobutyl-1-methylxanthine (IBMX), and 5 μg/mL insulin. Cells were maintained in this induction medium for 8 days, with fresh medium changes every 48 h. Finally, DIM-differentiated adipocytes were cultured for an additional 48 h in maintenance medium (DMEM + 5% FBS containing 5 μg/mL insulin, without dexamethasone or IBMX).

To evaluate the purity of DIM-differentiated adipocyte cultures, we performed real-time PCR to measure mRNA expression of the adipocyte-specific marker PPARγ, and markers associated with potential contaminating cell types, including PECAM1 for endothelium, CD45 for immune cells, FN1 for myofibroblasts, and SM22 for smooth muscle cells. PPARγ was robustly expressed in DIM-differentiated adipocytes, whereas markers of non-adipocytic lineages were negligible (all fold-change <0.03 vs PPARγ, p<0.05), indicating minimal contamination.

### 2.5 Fluorescence and electronic microscopy

DIM-differentiated adipocytes were cultured on 35 mm µ-Dishes (Ibidi, Munich, Germany) for 10 days, then separately stained with 200 nM MitoTracker™ Green (Invitrogen, Waltham, MA) to assess mitochondrial morphodynamic, 10 µM dihydroethidium (DHE; Invitrogen) to evaluate oxidative stress (Maneschi et al., 2012; Maneschi et al., 2016), and BODIPY™ 493/503 (Thermo Fisher Scientific, Waltham, MA) to investigate lipid storage. On the stained samples, instant images and timelapses were acquired by using DMI6000 microscope (Leica Camera AG, Wetzlar, Germany) equipped with a DFC350FX camera and a thermo-regulated incubation chamber (PeCon GmBH, Erbach, Germany) with controlled humidity and CO_2_ for cells maintenance. Additionally, electron microscopy was employed to evaluate mitochondrial cristae morphometry in DIM-differentiated adipocytes (n=6 for each experimental group). After being cultured with differentiation growth medium for 10 days in 60 mm dishes, cells were finally collected, pelleted by centrifugation and fixed in fixed in Karnowsky buffer and 1% osmium tetroxide and embedded in Epon 812 (EMS, Hatfield, PA). Ultrathin sections (70 nm) of DIM-differentiated adipocytes were prepared, stained with uranyl acetate and lead citrate, and observed at 80,000× magnification using a JEM 1010 transmission electron microscope (Jeol, Tokyo, Japan) at 80 kV, as previously described (Maneschi et al., 2016; Comeglio et al., 2018). Segmentation of mitochondrial cristae was carried out blindly using the iTEM image-analysis software (SIS), as previously described (Maneschi et al., 2016; Comeglio et al., 2018).

#### 2.5.1 Mitochondrial dynamics and morphology analysis

Mitochondrial morphodynamic properties were evaluated in DIM-differentiated adipocytes (n=6 for each experimental group) as indicators of mitochondrial functional status. All mitochondrial morphological measurements were performed under blind conditions to minimize potential bias. Mitochondrial length was assessed by manually segmenting individual mitochondria and restricting the analysis to regions of interest located at the cell periphery, while perinuclear areas were excluded. For mitochondrial length assessment, DIM-differentiated adipocytes were incubated with MitoTracker™ Green for 30 min at 37 °C in the dark. After staining, cells were washed with PBS and transferred into fresh culture medium before imaging. Images were captured through 63x/1.4NA oil-immersion objective. For each biological replicate, 13 to 20 individual DIM-induced adipocytes from 10 distinct microscopic fields were analyzed for each population, totalling approximately 3,000 total measurements of mitochondrial length per group. Morphometric analyses were performed using Fiji (ImageJ; https://imagej.net/software/fiji/) (Schindelin et al., 2012), and mitochondria were manually segmented to calculate average length per cell. Mitochondrial motility was analysed in live DIM-differentiated adipocytes performing 180 seconds timelapses (15 sec each acquisition). Cells stained with MitoTracker™ Green were imaged every 5 sec for the total duration of 180 sec under identical conditions.

The movement of individual mitochondria was tracked using the TrackMate plugin in Fiji (https://imagej.net/plugins/trackmate/) (Tinevez et al., 2017; Ershov et al., 2022). Velocity values were extracted and expressed as mean displacement rate (nm/s). Only mitochondria clearly visible and distinguishable throughout the time series were considered. Regions adjacent to the nucleus were excluded to avoid movement artifacts. A total of 13–20 cells from 10 randomly selected fields were analysed per cell lineage. Cristae morphology was evaluated by transmission electron microscopy (TEM). The surface area occupied by mitochondrial cristae was quantified and expressed as a percentage of total mitochondrial area, as previously described (Maneschi et al., 2013; Maneschi et al., 2016).

#### 2.5.2 Intracellular reactive oxygen species analysis

Intracellular superoxide production was evaluated blindly using dihydroethidium (DHE), a fluorogenic probe that intercalates into nuclear DNA upon oxidation to ethidium. DIM-differentiated adipocytes were incubated with DHE (10 μM in phenol red-free DMEM) at 37 °C for 30 min in the dark (RD+Veh, n=6; HFD+Veh, n=6; HFD + 0.3mg, n=5; HFD + 1.0mg, n=6; HFD + 5.0mg, n=6). After staining, cells were washed twice with warm PBS and maintained in fresh medium for live-cell imaging. Fluorescence timelapse microscopy was performed using a 63×/1.4NA oil-immersion objective. Timelapse acquisition was conducted over 180 sec, with one frame acquired every 15 sec, resulting in 13 total timepoints per field. For each biological replicate, 13–20 individual cells were analysed from 10 randomly selected fields per condition.

The oxidative response was quantified by measuring the intranuclear fluorescence intensity of DHE at the first (T_0_) and final (T_180_) timepoints of the timelapse series. The oxidative response was quantified by calculating the percentage increase in intranuclear DHE fluorescence intensity over time using the formula: ΔDHE (%) = [(F_t180_ – F_t0_)/F_t0_] × 100, where F_t0_ and F_t180_ represent the nuclear DHE fluorescence intensities at time 0 and 180 sec, respectively. This value reflects the percentage increase in oxidized DHE fluorescence within the nucleus and serves as an indirect index of intracellular superoxide generation, as described previously (Maneschi et al., 2013). Fluorescence measurements were performed in Fiji ImageJ Software using manual region-of-interest (ROI) selection for individual nuclei and background subtraction for each time point. To ensure consistency, all image acquisition settings (laser power, detector gain, pinhole size, offset) were kept constant across all samples.

#### 2.5.3 Oxygen consumption readouts ATP quantification and SDH activity

Intracellular ATP levels were quantified using the CellTiter-Glo^®^ Luminescent Cell Viability Assay (Promega, Madison, WI) following the manufacturer’s instructions (n=6 for each experimental group). DIM-differentiated adipocytes were seeded in white opaque 96-well plates at a density of 1×10^4^ cells per well and cultured under standard conditions. For each experimental group, each of the 6 biological replicates was analyzed in 6 technical replicates. At the endpoint, a volume of 125 μL CellTiter-Glo^®^ reagent was added to 3 wells, and the plates were incubated at room temperature for 10 min to stabilize the luminescent signal. Luminescence was measured using a Victor Nivo multilabel plate reader (Revvity, Waltham, MA), as previously described (Comeglio et al., 2018). ATP levels were normalized to the total protein content (µg) of each biological replicate. For each replicate, ATP measurements were performed in three wells and averaged, while total protein content was determined from three parallel wells seeded at the same density and treated identically, and the resulting mean protein value was used for normalization.

SDH activity was measured (n=6 for each experimental group) in cell lysates (50 µg total protein) incubated in phosphate buffer containing sodium azide, 2,6-dichlorophenolindophenol (DCPIP), sodium succinate, and phenazine methosulfate, as previously described (Kirby et al., 2007; Rapizzi et al., 2012). The decrease in absorbance due to DCPIP reduction was monitored at 600 nm using the Victor Nivo multilabel counter (Revvity). Enzymatic activity was calculated as the rate of DCPIP oxidation, normalized to total protein content and finally expressed as percent variation relative to control values (RD = 100% activity). The evaluation considered the peak point of the reaction curve.

#### 2.5.4 Lipid droplets analysis

The morphological characterization of intracellular lipid droplets in DIM-differentiated adipocytes was performed blindly on 2D fluorescent images acquired after BODIPY™ staining (RD+Veh, n=6; HFD+Veh, n=5; HFD + 0.3mg, n=5; HFD + 1.0mg, n=6; HFD + 5.0mg, n=6). Briefly, cells were incubated with BODIPY™ 493/503 for 15 min in the dark, fixed in 4% paraformaldehyde for 15 min at room temperature, and finally washed with PBS before acquisition. Fluorescent images were captured at 40× air magnification maintaining uniform acquisition parameters across all samples. For quantitative image analysis, single-plane (2D) images were processed using Fiji ImageJ Software. For visualization purposes, representative images have been processed using an ImageJ lookup table (LUT) to enhance lipid droplet contrast and reduce background signal. It is important to note that our experimental model consists of DIM-induced preadipocytes that were not supplemented with exogenous fatty acids (e.g. palmitate or oleate) or other lipid-loading agents commonly used *in vitro* to promote extensive lipid accumulation. Under these conditions cells undergo adipogenic differentiation, albeit only in a manner partially comparable to mature adipocytes isolated directly from adipose tissue. Accordingly, lipid storage at this stage is typically characterized by small lipid droplets, rather than the large unilocular droplets observed in adipocytes from the tissue or lipid-loaded adipocytes (Heid et al., 2014).

Lipid droplet segmentation and quantification were performed using the Lipid Droplets Tool macro (https://github.com/MontpellierRessourcesImagerie/imagej_macros_and_scripts/wiki/Lipid-Droplets-Tool) (Montanari et al., 2019; Ali et al., 2023), developed by the Institut de Génétique Moléculaire de Montpellier (CNRS, France). Prior to analysis, images were converted to 8-bit grayscale and thresholded using an automated method (Otsu, 1979) or manually adjusted for optimal droplet detection. A watershed separation algorithm was applied to resolve closely packed droplets. For each detected lipid droplet, individual morphometric parameters were extracted, including projected area and Feret diameters (minimum and maximum). An estimate of lipid droplet volume was calculated using the simplified spherical formula: V=(π/6)·[(minFeret+maxFeret)/2]³, where minFeret and maxFeret, refer to the minimum and maximum diameters of each object, respectively. The formula is derived from the standard expression for the volume of a sphere V=(π/6)·D³, where D=(minFeret+maxFeret)/2 (Corbisier et al., 2015; Attota and Liu, 2016; Pei et al., 2021). These values represent an estimated volume based on 2D morphometric parameters rather than absolute 3D volumes. A range of 13–20 cells from 10 randomly selected fields across biological replicates were analysed, collecting a minimum number of 2,500 lipid droplets. Data were compiled and statistically analysed to assess differences in lipid droplet size and distribution among experimental groups.

### 2.6 RNA extraction and biomarkers expression analysis

Total RNA was extracted from DIM-differentiated adipocytes (n=6 per experimental group) and from VAT homogenates. RNA isolation was performed using the RNeasy Mini Kit (Qiagen, Hilden, Germany) according to the manufacturer’s protocol for cultured cells, including supplementation of the lysis buffer with 1% β-mercaptoethanol and on-column DNase digestion to eliminate residual genomic DNA. For the available VAT homogenates (RD+Veh, n=6; HFD+Veh, n=7; HFD + 0.3 mg, n=7; HFD + 1.0 mg, n=7; HFD + 5.0 mg, n=7), samples were initially processed with TRIzol reagent (Life Technologies, Paisley, UK) prior to purification. First-strand cDNA was synthesized using the iScript™ cDNA Synthesis Kit (Bio-Rad Laboratories, Hercules, CA), and semiquantitative real-time PCR (qRT-PCR) was carried out using the CFX Duet Real-Time PCR Detection System (Bio-Rad) with SsoAdvanced™ Universal SYBR^®^ Green Supermix (Bio-Rad).

Primer sequences for rabbit target genes were designed based on NCBI GenBank (http://www.ncbi.nlm.nih.gov) and Ensemble Genome (https://www.ensembl.org/index.html) database entries and were validated for efficiency and specificity.

The 18S ribosomal RNA subunit was used as the housekeeping gene for the relative quantitation of the target genes based on the comparative threshold cycle (Ct) 2^−ΔΔCt^ method (Livak and Schmittgen, 2001), with some modifications.

In detail, we used the vehicle-treated control group as the calibrator in each analysis, so that the calculations would provide the fold-change of the other groups relative to controls. The 18S ribosomal RNA subunit was quantified using primers designed based on the rabbit transcript ENSOCUT00000030594.2:

- Forward - 5′-GGCCGTTCTTAGTTGGTGGA-3′
- Reverse - 5′-AGCATGCCAGAGTCTCGTTC-3′

The amplification efficiency for 18S was determined to be greater than 94%.

### 2.7 Statistical analysis

Data are expressed as mean ± standard error of the mean (SEM). Only biological replicates were included in the statistical analyses, with each animal contributing a single mean value, irrespective of the number of technical measurements performed. For each experimental group, the value was calculated as the average of the individual animal means (n=6 per group) and used for comparisons. With this sample size, the study provides approximately 80% power to detect large, biologically meaningful effects (Cohen’s f ≈ 0.40), consistent with prior preclinical reports. Due to the relatively small sample size per group (n=6) and potential deviations from normality and equal variance, overall differences were assessed using the one-way non-parametric Kruskal-Wallis ANOVA. Pairwise Mann-Whitney U tests were performed for exploratory comparisons between groups. These comparisons are interpreted in the context of the overall Kruskal-Wallis results, providing a balance between meaningful group-level analysis and the limitations of our sample size.

Pearson’s product-moment correlation coefficient (Bravais-Pearson correlation) was used to assess linear associations between variables. A p value <0.05 was considered statistically significant.

Sample sizes, test types, and adjusted p-values are reported in the corresponding figure legends and in the results section. Statistical analyses were performed using either open-source R (R Core Team, downloadable at https://www.r-project.org/) or Statistical Package for the Social Sciences (v.29.0.2; IBM SPSS, Armonk, NY).

## 3 Results

### 3.1 Clinical and biochemical data

**Figure 1:**
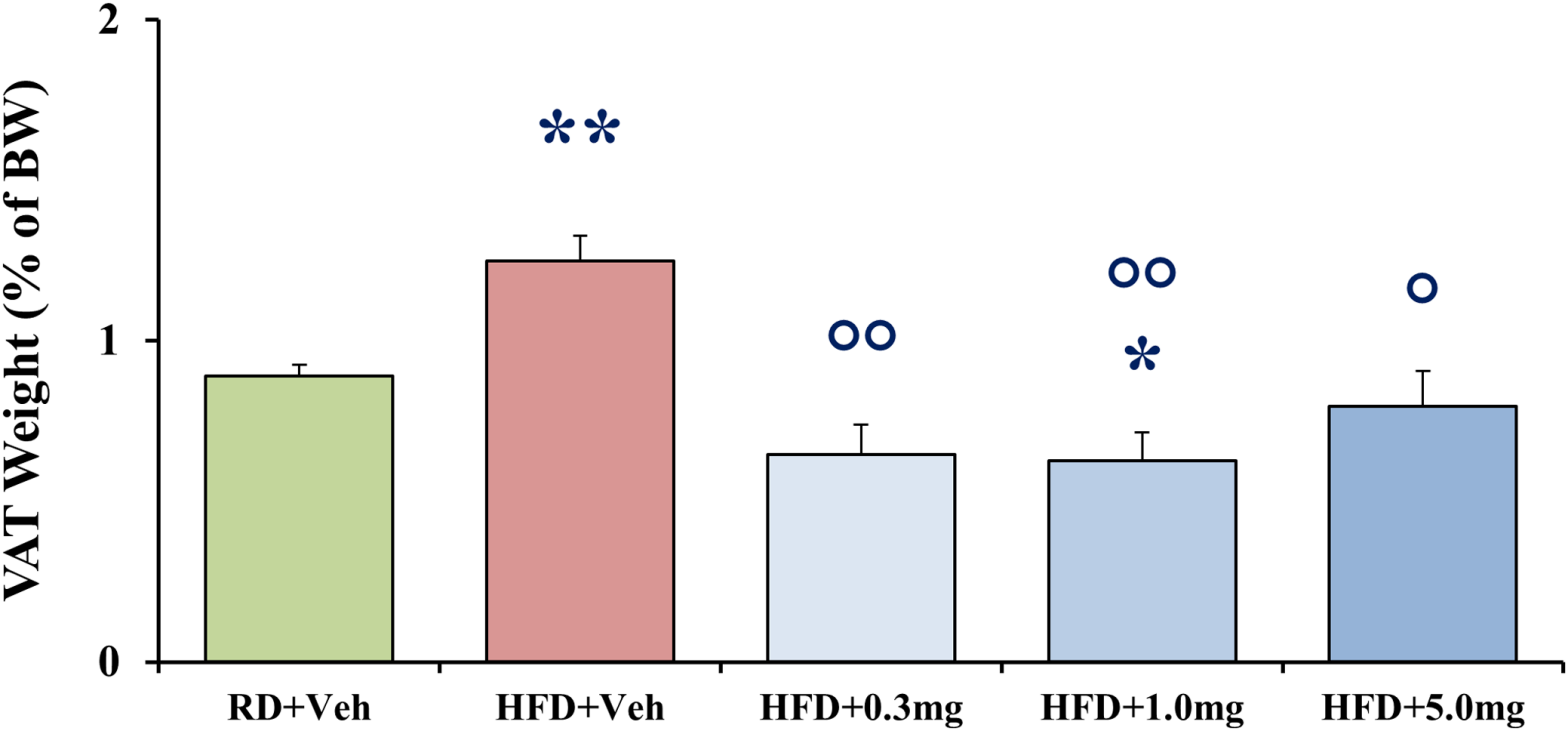
Selvigaltin effects on VAT weights in HFD-induced Mets model. VAT weight at the end of treatment is expressed as a percentage of total body weight and reported as mean ± SEM (n=6 for each experimental group). Statistical analysis for group comparisons was performed using one-way non-parametric Kruskal-Wallis ANOVA followed by *post hoc* Mann-Whitney analysis. * p<0.05, ** p<0.01 vs RD+Veh; ° p<0.05, °° p<0.01 vs HFD+Veh.

In this study we found that rabbits fed a HFD exhibited a marked deterioration in their metabolic profile compared to those on a regular diet (**Supplementary Table ST1**), in perfect agreement with previous observations in this animal model (Filippi et al., 2009; Maneschi et al., 2013; Vignozzi et al., 2014; Comeglio et al., 2018; Comeglio et al., 2021; Sarchielli et al., 2021). Accordingly, **Figure 1** shows a significant increase in visceral adipose tissue (VAT) mass, expressed as a percentage of total body weight, from in HFD animals, as compared to regular diet (RD) rabbits. This VAT expansion was completely normalized by *in vivo* selvigaltin dosing at all doses tested (p<0.01 vs HFD + 0.3mg and HFD + 1.0mg, p<0.05 vs HFD + 5.0mg, all compared to HFD+Veh; **Figure 1**), in agreement with what was reported in a larger series of animals (Comeglio et al., 2024).

### 3.2 Mitochondrial morphology in visceral DIM-differentiated adipocytes

**Figure 2:**
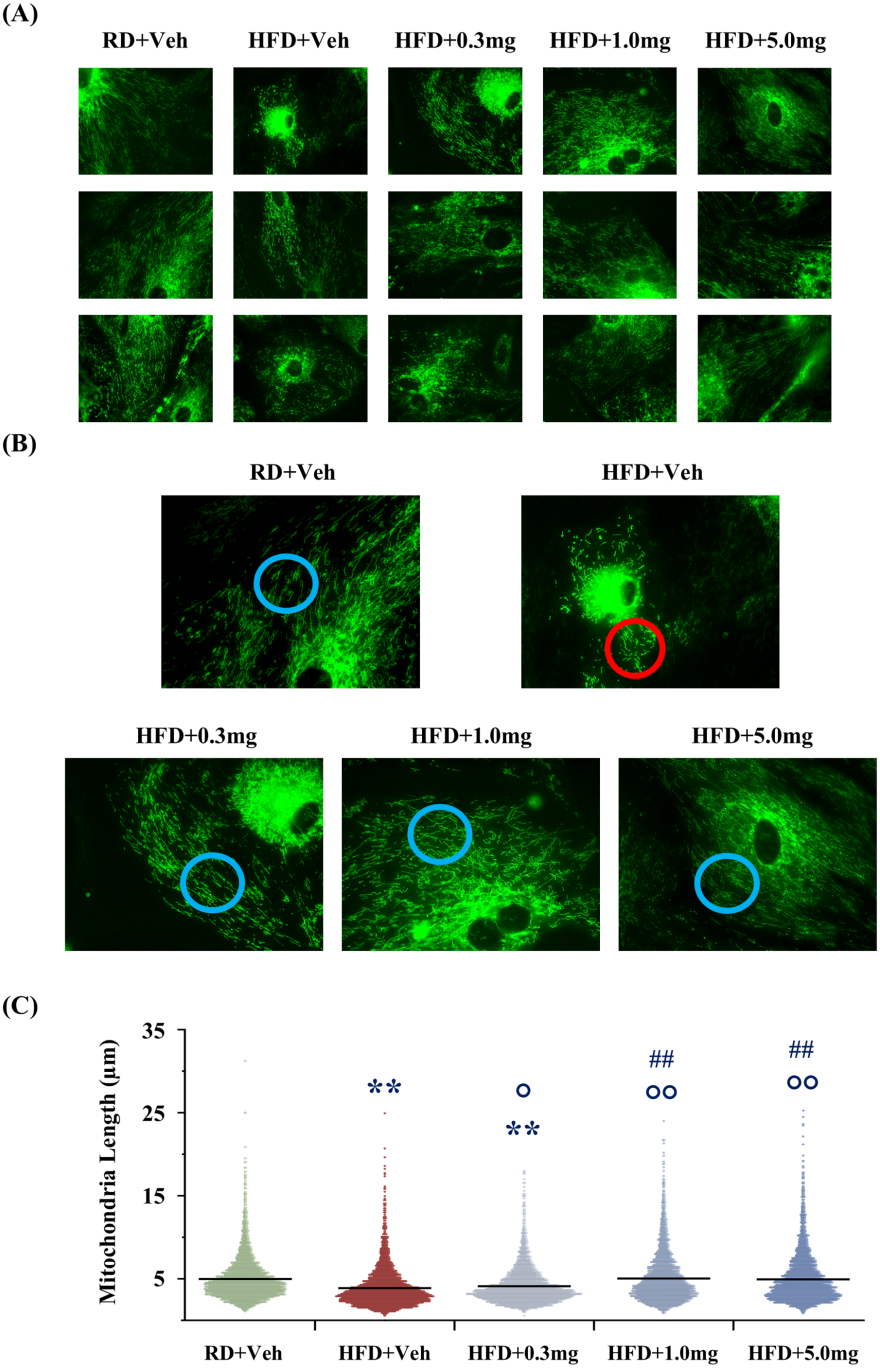
Mitochondrial fluorescence imaging with MitoTracker in DIM-differentiated adipocytes. Panels **(A, B)** show representative images of cells labeled with MitoTracker, with Panel **(B)** presenting a magnified view of one of the samples for each group, to better highlight the mitochondrial structures (red circle for HFD+Veh). These images were selected from multiple independent microscopic fields to accurately reflect the overall population phenotype. Panel **(C)** reports the average and the distribution of mitochondrial lengths for each experimental group. Data are shown as mean+SEM. Despite the violin density scatter plot reporting all measurements, the mean for each biological replicate (n=6 for each experimental group) has been considered for statistical purposes: the black bar in the plot indicates the average mitochondrial length derived from the means of each experimental group. Statistical analysis for group comparisons was performed using one-way non-parametric Kruskal-Wallis ANOVA followed by *post hoc* Mann-Whitney analysis. **p<0.01 vs RD+Veh; °p<0.05, °°p<0.01 vs HFD+Veh; ^##^p<0.01 vs HFD + 0.3mg.

To evaluate potential phenotypic changes in DIM-differentiated adipocytes following *in vivo* selvigaltin treatment, we examined mitochondrial morphology via fluorescence microscopy. Our observations (**Figures 2A, B**) revealed distinct alterations in the mitochondrial network configuration in response to HFD feeding. In cultures derived from HFD, the mitochondrial network displayed a loss of continuity, appearing primarily as short, isolated fragments rather than an interconnected network (**Figures 2A, B**). While these 2D profiles suggest an increased presence of fragmented mitochondria, further 3D structural analyses are warranted to fully characterize the network architecture.

In contrast, *in vivo* selvigaltin-treated groups exhibited elongated and interconnected mitochondrial networks, closely resembling the morphology of RD DIM-differentiated adipocytes, as shown in the high magnification images in **Figure 2** (panel B, blue circles). Specifically, DIM-induced adipocytes from HFD+Veh animals displayed a significant reduction in mitochondrial length (3.87 ± 0.07 µm; p<0.01) compared to RD+Veh (4.98 ± 0.04 µm; **Figure 2C**), consistent with a fragmented mitochondrial network typically associated with metabolic stress and impaired bioenergetics.

Selvigaltin treatment appeared to normalize the mitochondrial architecture, with a partial recovery observed using 0.3 mg/kg (4.10 ± 0.06 µm; p<0.05 vs HFD+Veh), whereas doses of 1.0 and 5.0 mg/kg normalized mitochondrial length to RD+Veh levels (5.03 ± 0.02 and 4.93 ± 0.06 µm, respectively; both p<0.01 vs HFD+Veh; **Figure 2C**). The violin density scatter plot shows that, in the HFD+Veh group, the majority of mitochondria clustered at very short lengths, predominantly between 1 and 2 µm, indicating a fragmented and bioenergetically compromised mitochondrial population.

Notably, this group also exhibited a marked depletion of mitochondria in the intermediate range (5-10 µm), a length category abundantly represented in controls and associated with metabolically competent, elongated mitochondrial networks.

Selvigaltin treatment progressively and significantly reshaped the distribution profile in a dose-dependent manner. Using the 5.0 mg/kg dose of selvigaltin, a clear restoration of the full spectrum of mitochondrial lengths was observed, including the reappearance of the intermediate 5-10 µm population, thus suggesting a functional recovery of mitochondrial dynamics. The distribution pattern in the higher-dose groups closely approximated that of RD DIM-differentiated adipocytes, confirming the re-establishment of a physiologically heterogeneous mitochondrial network.

### 3.3 Mitochondrial ultrastructure and cristae architecture in visceral DIM-differentiated adipocytes

**Figure 3:**
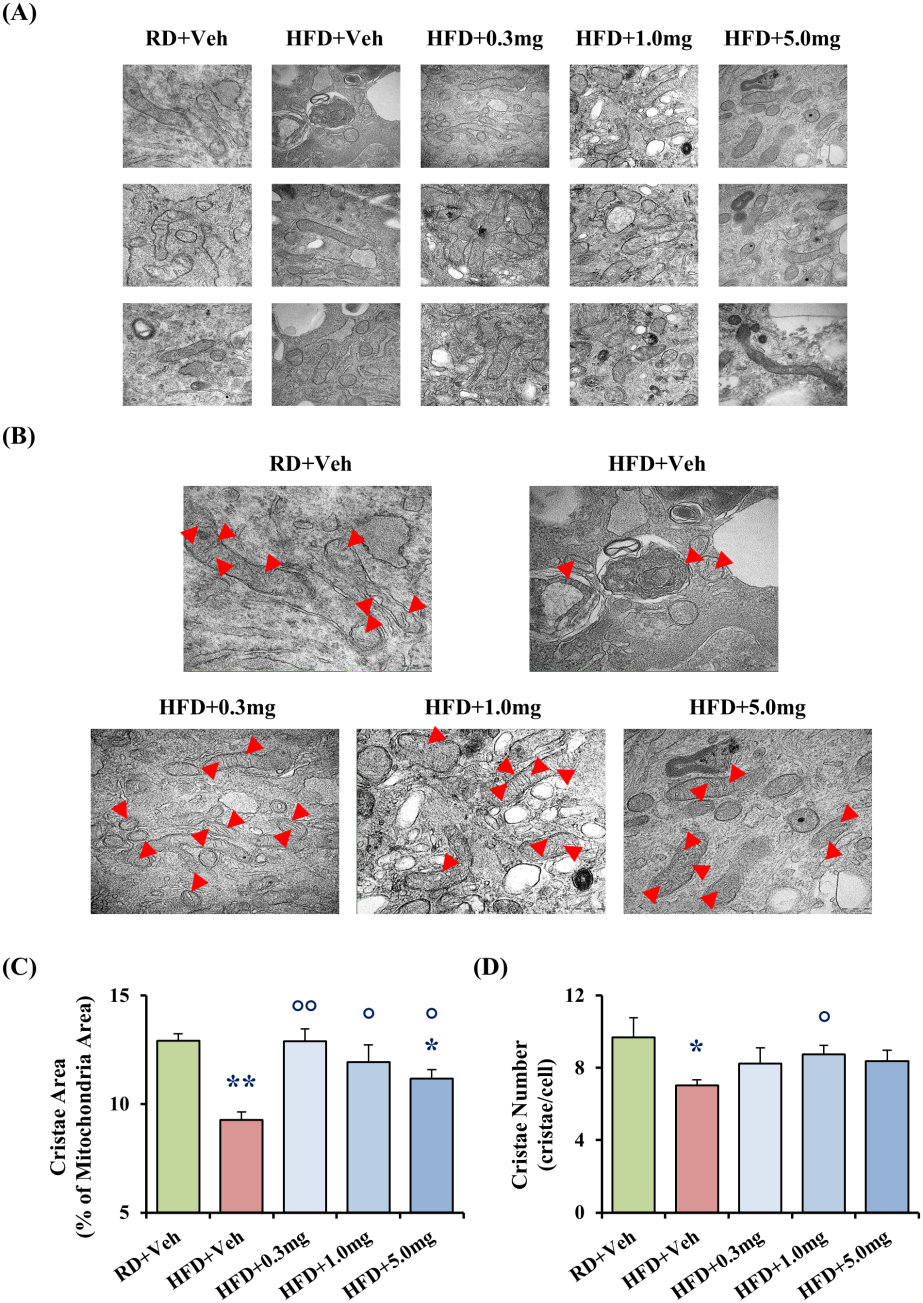
Mitochondrial ultrastructure imaging with TEM in DIM-differentiated adipocytes. Panels **(A)** and **(B)** show representative images of mitochondrial ultrastructure, with Panel **(B)** presenting a magnified view to better highlight the mitochondrial cristae (red arrows). These images were selected from multiple independent microscopic fields to accurately reflect the overall population phenotype. Panel **(C)** reports the average mitochondrial cristae area as percentage of the mitochondrial area. Panel **(D)** shows the average per cell number of cristae within each group. Data are shown as mean+SEM (n=6 for each experimental group). Statistical analysis for group comparisons was performed using one-way non-parametric Kruskal-Wallis ANOVA followed by *post hoc* Mann-Whitney analysis. *p<0.05, **p<0.01, vs RD+Veh; °p<0.05, °°p<0.01, vs HFD+Veh.

Transmission electron microscopy (TEM) was used to evaluate mitochondrial ultrastructure and cristae organization in DIM-differentiated adipocytes (**Figure 3**).

As shown in representative TEM micrographs (**Figure 3A**), DIM-induced adipocytes from HFD+Veh animals displayed swollen and rounded mitochondria with reduced internal compartmentalization, consistent with disrupted cristae morphology. This contrasts with the elongated and electron-dense mitochondria observed in RD+Veh animals, which showed well-organized cristae structures (**Figure 3A**, left column). High-magnification images (**Figure 3B**) further highlighted these alterations, showing sparse and irregular cristae in HFD+Veh mitochondria, while all doses of *in vivo* selvigaltin treatments restored a more compact and layered cristae pattern. Quantitative morphometric analysis confirmed these visual findings. As shown in **Figure 3C**, the mean cristae area was significantly reduced in HFD+Veh DIM-differentiated adipocytes (9.28 ± 0.37%) compared to RD+Veh (12.91 ± 0.32%; p<0.01).

All selvigaltin treatment doses significantly increased cristae area (HFD + 0.3mg, p<0.01 vs HFD+Veh; HFD + 1.0mg and HFD + 5.0mg, both p<0.05 vs HFD+Veh), restoring it to RD levels, with the best effect at 0.3mg/kg and 1.0 mg/kg. The highest dose (5 mg/kg) maintained this recovery, with mean values of around 11.16%, in fact almost normalizing the data.

In parallel, the analysis of mitochondrial cristae number (**Figure 3D**) revealed a similar trend, with the mean cristae number per cell reduced in HFD+Veh animals (7.01 ± 0.30) compared to RD+Veh (9.70 ± 1.08; p<0.05) and exhibiting a consistent upward trend across all selvigaltin-treated groups. Even though not always reaching statistical significance, selvigaltin groups ranged from 8.24 to 8.74, closely approximating RD cristae number levels, indicating a substantial restoration of the inner membrane structure.

### 3.4 Mitochondrial dynamics in visceral DIM-differentiated adipocytes

To further investigate the impact of Gal-3 inhibition on mitochondrial integrity, both structural and dynamic parameters were evaluated in DIM-differentiated adipocytes.

**Figure 4:**
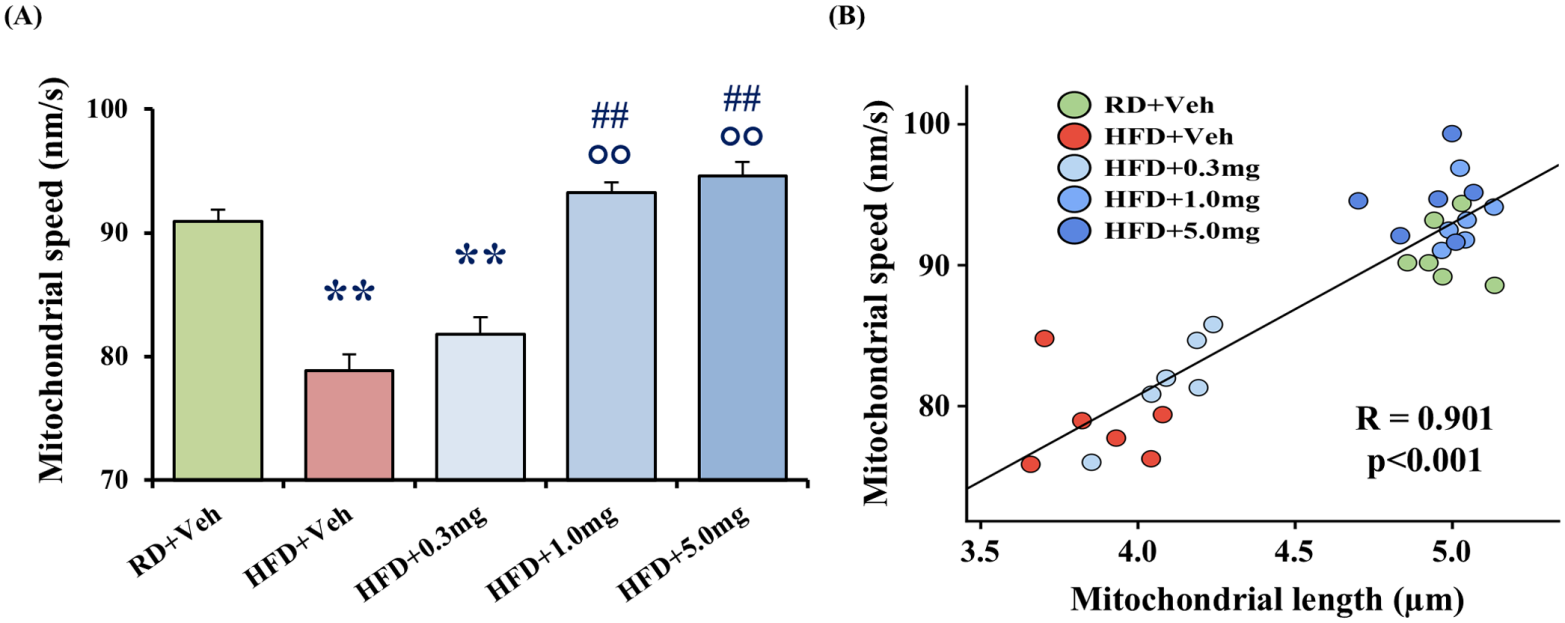
Mitochondrial dynamics evaluation in DIM-differentiated adipocytes. Panel **(A)** shows the quantification of mitochondrial speed. Panel **(B)** represents the positive Bravais-Pearson correlation between mitochondrial speed and length reported as fold change vs RD+Veh. All data are reported as mean ± SEM (n=6 for each experimental group). Statistical analysis was performed using one-way non-parametric Kruskal-Wallis ANOVA followed by *post hoc* Mann-Whitney analysis [panel **(A)**] for group comparisons, and Bravais-Pearson correlation [panel **(B)**]. **p<0.01 vs RD+Veh; °°p<0.01 vs HFD+Veh; ^##^p<0.01 vs HFD + 0.3mg.

As previously shown (**Figures 2A–C**), mitochondria from HFD+Veh animals displayed a fragmented morphology with significantly reduced length, indicative of impaired mitochondrial fusion and turnover capacity. To assess mitochondrial dynamics, live-cell imaging and timelapse analysis were used to quantify mitochondrial motility, expressed as average displacement speed (nm/s) (**Figure 4A**).

HFD dieting significantly reduced mitochondrial speed (78.84 ± 1.32 nm/s) compared to RD DIM-differentiated adipocytes (90.94 ± 0.95 nm/s; p<0.01), indicating a loss of mitochondrial plasticity and trafficking. Representative timelapse videos are available as **Supplementary Material** (Mito_<*name of group*> AVI files).

Moreover, from the timelapse recordings, an increase in background signal was also appreciable in the HFD+Veh group, accompanied by a concomitant loss of MitoTracker signal specifically in the mitochondria, suggesting mitochondrial membrane damage upon exposure to UV-induced phototoxic stress. Notably, *in vivo* selvigaltin treatments showed a robust, dose-dependent increase in mitochondrial motility. Although the lowest dose (0.3 mg/kg) failed to induce a significant and consistent recovery in mitochondrial speed compared to controls, it still showed a slight improvement (81.77 ± 1.40 nm/s) compared to the HFD+Veh group.

Considering the other treatment groups, mitochondrial speed was restored to RD levels and significantly increased, compared to HFD+Veh, using 1.0 and 5.0 mg/kg doses (93.26 ± 0.85 and 94.58 ± 1.12 nm/s, respectively; both p<0.01 vs HFD+Veh), suggesting enhanced organelle turnover and improved bioenergetic dynamics. Moreover, Bravais-Pearson correlation analysis revealed a strong positive relationship (R = 0.901, p<0.001) between mitochondrial length and mitochondrial speed across experimental groups, as shown in **Figure 4B**.

### 3.5 Mitochondrial oxidative capacity in visceral DIM-differentiated adipocytes

**Figure 5:**
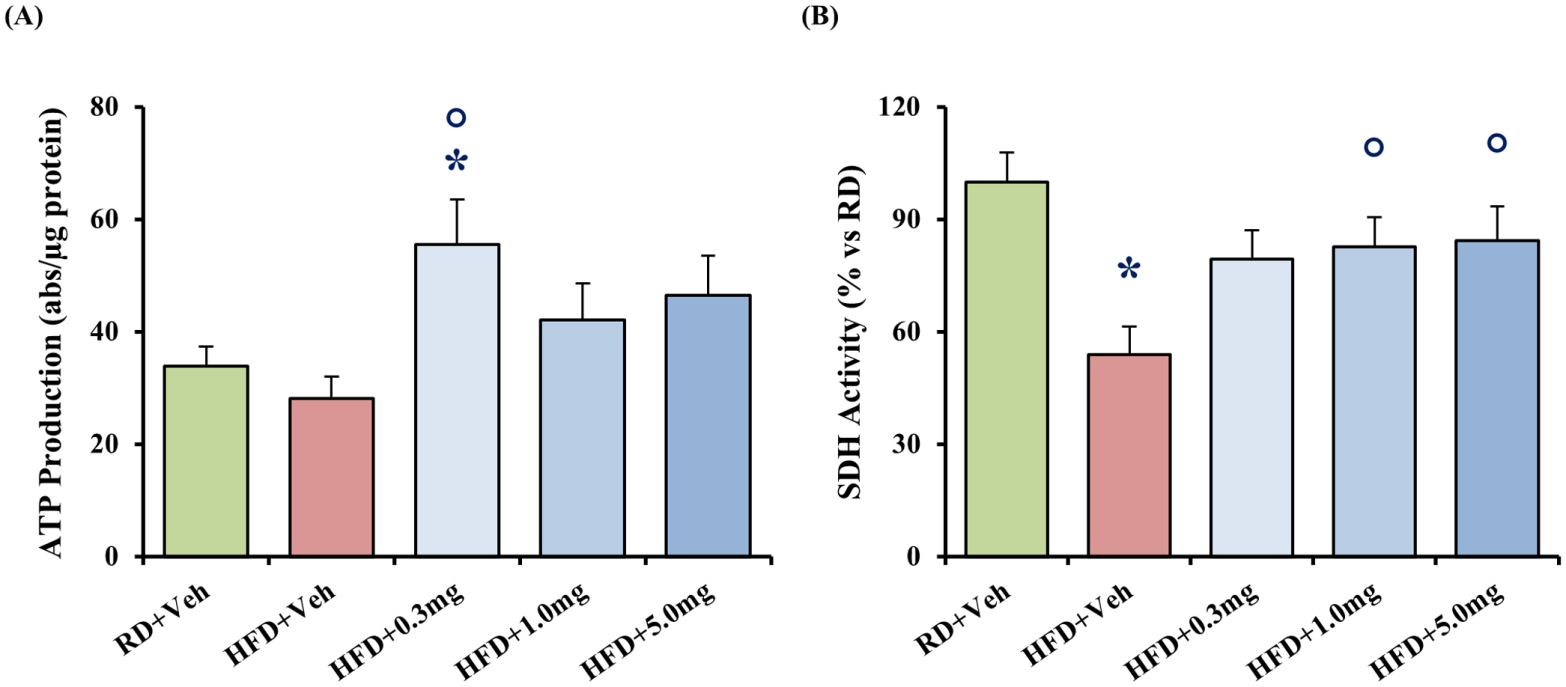
Oxygen consumption readouts in DIM-differentiated adipocytes. Panel **(A)** reports ATP production levels. ATP data was normalized over protein content (µg) of each sample. Panel **(B)** shows SDH activity. All data are reported as mean ± SEM (n=6 for each experimental group). Statistical analysis for group comparisons was performed using one-way non-parametric Kruskal-Wallis ANOVA followed by *post hoc* Mann-Whitney analysis. *p<0.05 vs RD+Veh; °p<0.05 vs HFD+Veh.

To assess mitochondrial respiratory function, two complementary surrogate parameters of oxygen consumption were evaluated, namely ATP production and succinate dehydrogenase (SDH) activity. As shown in **Figure 5A**, ATP production was slightly reduced in HFD+Veh DIM-differentiated adipocytes, compared to RD+Veh (28.11 ± 3.93 vs 33.96 ± 3.44 absorbance/μg protein, respectively), suggesting a moderately impaired oxidative phosphorylation following HFD exposure. *In vivo* selvigaltin treatments showed an increasing trend across all doses, with the highest value observed at 0.3 mg/kg (55.51 ± 8.04; p<0.05 vs both RD+Veh and HFD+Veh). Interestingly, ATP levels in the other selvigaltin-treated groups also exceeded those observed in control animals, albeit without reaching statistical significance.

Similarly, SDH activity, an established indicator of electron transport chain integrity and tricarboxylic acid (TCA) cycle flux, was markedly reduced in HFD+Veh cells (54.01 ± 7.35% vs RD set at 100%, p<0.05) (**Figure 5B**).

All selvigaltin-treated groups showed increased SDH activity compared to HFD+Veh, with values at 0.3 mg/kg (79.38 ± 7.72%), 1.0 mg/kg (82.74 ± 7.78%; p<0.05 vs HFD+Veh), and 5.0 mg/kg (84.30 ± 9.15%; p<0.05 vs HFD+Veh), suggesting a consistent improvement in mitochondrial oxidative metabolism.

### 3.6 ROS accumulation in visceral DIM-differentiated adipocytes

To investigate the effects of the Gal-3 inhibition on intracellular oxidative stress, ROS levels were evaluated in DIM-differentiated adipocytes using DHE fluorescence imaging. Representative timelapse videos are available as **Supplementary Material** (ROS_<*name of group*> AVI files).

**Figure 6:**
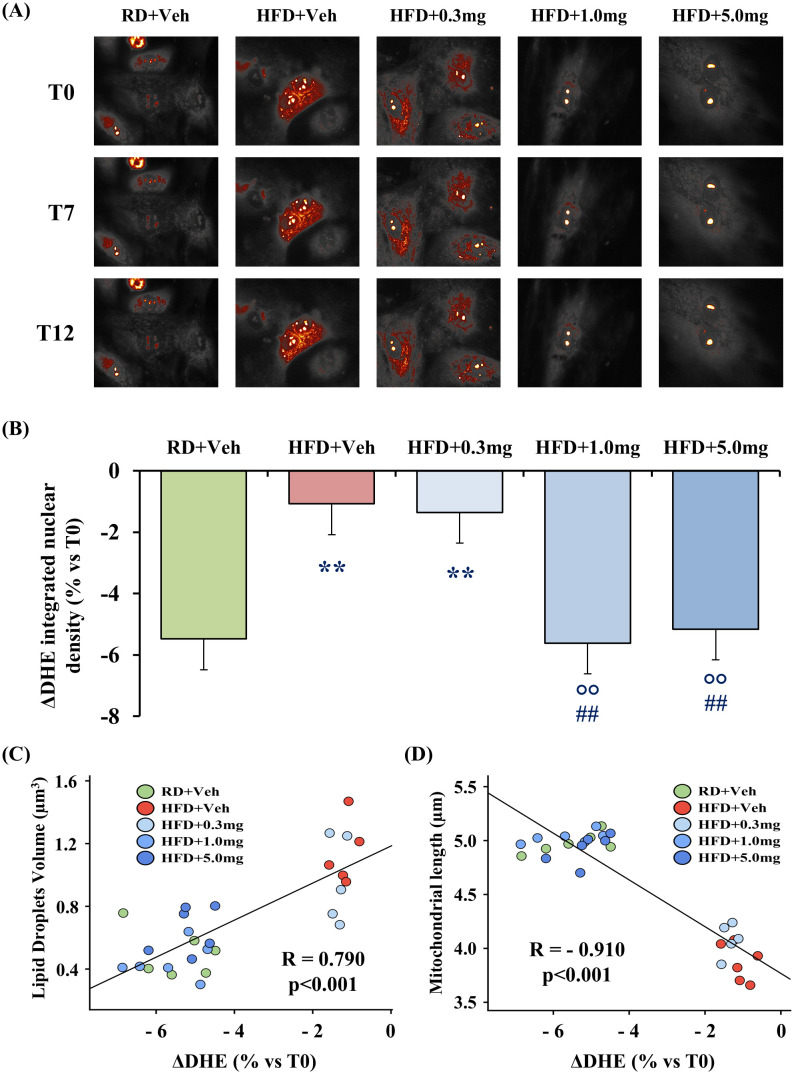
ROS fluorescence imaging with DHE in DIM-differentiated adipocytes. Panel **(A)** shows representative qualitative images of DHE acquisition signal from the timelapses for each group (T_0_ = 0 sec, T_7_ = 90 sec, T_12_ = 180 sec). Panel **(B)** shows ΔDHE integrated nuclear density (% vs T_0_) quantification. Panel **(C, D)** report, as fold change vs RD+Veh, the Bravais-Pearson correlation between ΔDHE integrated nuclear density and lipid droplets volume [panel **(C)**] or mitochondrial length [panel **(D)**]. All data are reported as mean ± SEM (RD+Veh, n=6; HFD+Veh, n=6; HFD + 0.3mg, n=5; HFD + 1.0mg, n=6; HFD + 5.0mg, n=6). Statistical analysis was performed using one-way non-parametric Kruskal-Wallis ANOVA followed by *post hoc* Mann-Whitney analysis [panel **(B)**] for group comparisons, and Bravais-Pearson correlation [panels **(C, D)**]. **p<0.01 vs RD+Veh; °°p<0.01 vs HFD+Veh; ^##^p<0.01 vs HFD + 0.3mg.

The timelapse images (**Figure 6A**) revealed a progressive increase in the nuclear DHE signal in HFD+Veh cells, suggestive of enhanced superoxide accumulation and redox imbalance. In contrast, cells from selvigaltin-treated animals, particularly at higher doses, maintained a low-intensity DHE signal over time, comparable to RD+Veh DIM-induced adipocytes.

Quantitative analysis of DHE fluorescence dynamics, shown as ΔDHE signal variation from T_0_ to T_180_, confirmed these visual findings (**Figure 6B**). HFD+Veh DIM-differentiated adipocytes exhibited a significant continuance in time in nuclear ROS levels (-1.08 ± 0.14%), compared to RD+Veh (-5.48 ± 0.37%; p<0.01 vs HFD+Veh). *In vivo* selvigaltin treatments led to marked clearances of ROS accumulation at most doses. Whereas the 0.3 mg/kg group showed an attenuation (-1.35 ± 0.08%) similar to HFD+Veh, higher doses completely abolished the oxidative response, normalizing ΔDHE values (-5.62 ± 0.36% at 1.0 mg/kg and -5.16 ± 0.25% at 5.0 mg/kg; both p<0.01 vs HFD+Veh).

To further explore the mechanistic relationships between oxidative stress and cellular phenotype, correlation analyses were performed. As shown in **Figure 6C**, nuclear ΔDHE signals significantly and positively correlated with lipid droplet volumes (R = 0.790; p<0.001), supporting the notion that excessive lipid accumulation promotes intracellular oxidative stress.

Conversely, a strongly significant negative correlation was observed between ΔDHE levels and mitochondrial lengths (R=-0.910, p<0.001; **Figure 6D**).

### 3.7 Lipid droplet morphology and lipid storage in visceral DIM-differentiated adipocytes

**Figure 7:**
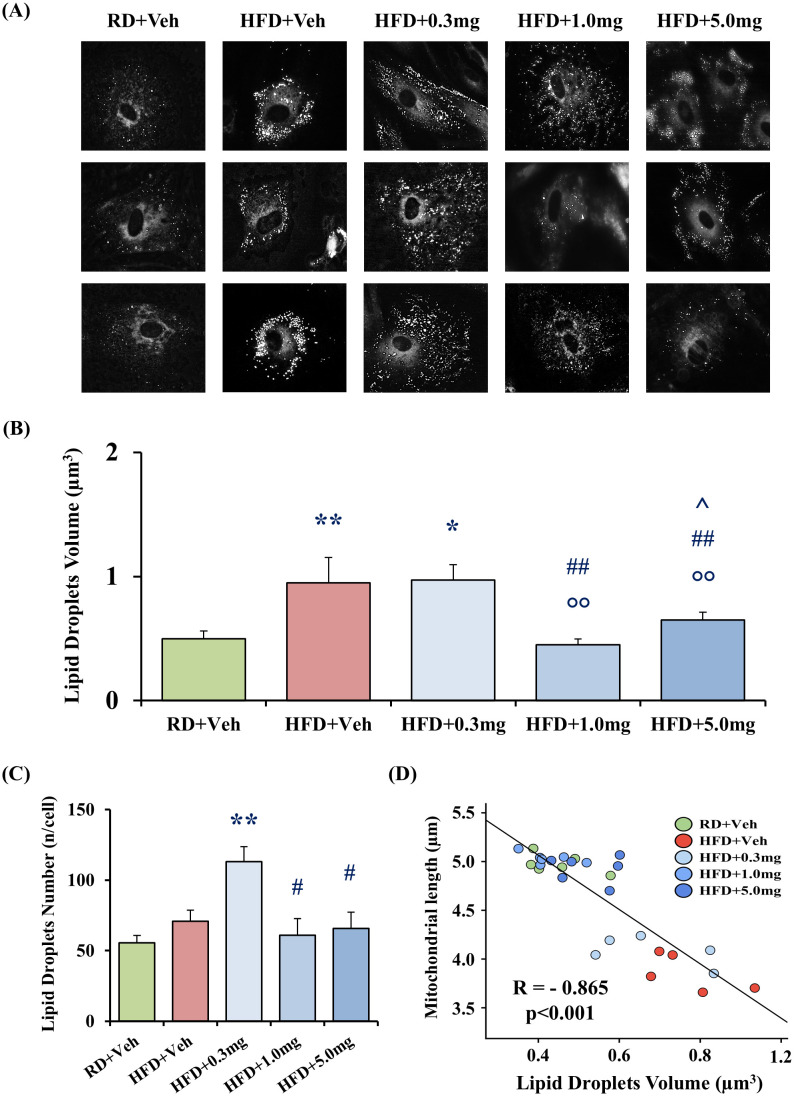
LD fluorescence imaging with BODIPY™ in DIM-differentiated adipocytes. Panel **(A)** shows representative images of BODIPY™ acquisition for each group processed using an ImageJ lookup table (LUT) to enhance lipid droplet contrast and reduce background signal. These images were selected from multiple independent microscopic fields to accurately reflect the overall population phenotype. Panel **(B, C)** show the quantification of LD volume and number per cell, respectively. Panel **(D)** reports the Bravais-Pearson negative correlation between LD volumes and mitochondrial length. All data are shown as mean ± SEM [(RD+Veh, n=6; HFD+Veh, n=5; HFD + 0.3mg, n=5; HFD + 1.0mg, n=6; HFD + 5.0mg, n=6)]. Statistical analysis was performed using one-way non-parametric Kruskal-Wallis ANOVA followed by *post hoc* Mann-Whitney analysis [panels **(B, C)**] for group comparisons, and Bravais-Pearson correlation [panel **(D)**]. *p<0.05, **p<0.01 vs RD+Veh; °°p<0.01 vs HFD+Veh; ^#^p<0.05, ^##^p<0.01 vs HFD + 0.3mg; ^p<0.05 vs HFD + 1.0mg.

To assess the impact of Gal-3 inhibition on lipid storage, BODIPY™ fluorescence imaging was performed in DIM-differentiated adipocytes to evaluate lipid droplet (LD) morphology, size, and abundance (**Figures 7A–C**). Rather than absolute 3D volumes, droplet size values represent an estimated volume calculated from 2D morphometric parameters. Cells from HFD+Veh animals exhibited an increase in LD accumulation, with apparently larger, coalescent droplets occupying a substantial portion of the cytoplasm. In contrast, RD+Veh cells displayed a fine and homogeneous distribution of smaller-sized droplets. Selvigaltin treatment markedly altered this phenotype, particularly at 1.0 and 5.0 mg/kg, where a restoration of ostensibly smaller, more evenly distributed lipid droplets was observed (**Figure 7A**).

Quantitative image analysis confirmed these morphological changes, as shown in **Figure 7B**, with LD volume significantly elevated in HFD+Veh cells (0.95 ± 0.20 μm³) compared to RD+Veh DIM-differentiated adipocytes (0.50 ± 0.06 μm³, p<0.01). *In vivo* selvigaltin treatments induced a dose-dependent reduction in LD volume, with values normalized and significant effects observed at 1.0 mg/kg and 5.0 mg/kg (both p<0.01 vs HFD+Veh).

As shown in **Figure 7C**, a moderate increase in the number of lipid droplets per cell has been detected in the HFD+Veh group (70.8 ± 7.9), compared to controls.

Interestingly, the LDs per cell increased significantly in the 0.3 mg/kg DIM-induced adipocytes (113.0 ± 10.7), compared to the other groups (at least p<0.05 vs all other groups).

Although not significantly, selvigaltin treatments showed a trend of normalization for this parameter at higher doses (60.8 ± 11.7 and 65.8 ± 11.6 for 1.0 and 5.0 mg/kg, respectively). Finally, a strong inverse correlation was observed between LD volume and mitochondrial length (**Figure 7D**; R=-0.865, p<0.001).

### 3.8 mRNA expression of genes involved in mitochondrial function, lipid storage, and inflammation

As shown in **Table 1**, several genes involved in mitochondrial structure and biogenesis were analyzed in mRNA extracted from DIM-differentiated adipocytes. *FIS1*, *MFN1*, *OPA1*, and *TFAM* mRNA expressions, slightly increased in the HFD+Veh group compared to controls, were significantly downregulated in the selvigaltin-treated groups. Similarly, lipid handling markers *PLIN1* and *SNAP23* mRNA expressions were elevated in HFD+Veh DIM-differentiated adipocytes vs RD+Veh and reduced in the selvigaltin-treated groups. Additionally, *BMP4* and *BMP7*, two key regulators of adipocyte differentiation, showed mRNA expression decreases in selvigaltin-treated animals, compared to HFD+Veh, therefore suggesting a restoration of pro-adipogenic transcriptional programs. Gal-3 inhibition was also associated with a broad transcriptional suppression of inflammatory genes. In particular, HFD increased all canonical inflammatory markers (COX2, IL-1β, IL-6, TLR2, TLR4, TNFα) mRNA expression, whereas selvigaltin treatment suppressed this upregulation at all doses.

**Table 1:** Biomarkers mRNA expression in DIM-differentiated adipocytes.

Mitochondrial markers	RD+ Veh	HFD+ Veh	Sig.	HFD+0.3mg	Sig.	HFD+1.0mg	Sig.	HFD+5.0mg	Sig.
*FIS1*	1.00 ± 0.06	1.13 ± 0.10		0.86 ± 0.07	°	0.95 ± 0.06		0.98 ± 0.05	
*MFN1*	1.00 ± 0.05	1.09 ± 0.05		0.83 ± 0.05	°°	0.94 ± 0.09		0.92 ± 0.03	°
*MFN2*	1.00 ± 0.07	1.06 ± 0.11		0.52 ± 0.05	** °°	0.65 ± 0.08	* °	0.56 ± 0.09	** °
*OPA1*	1.00 ± 0.10	1.10 ± 0.09		0.89 ± 0.07		0.92 ± 0.11		0.79 ± 0.04	°
*TFAM*	1.00 ± 0.16	1.28 ± 0.17		0.64 ± 0.07	°	0.92 ± 0.18		0.80 ± 0.07	°

LD storage Markers	RD+Veh	HFD+Veh	Sig.	HFD+0.3mg	Sig.	HFD+1.0mg	Sig.	HFD+5.0mg	Sig.
*PLIN1*	1.00 ± 0.19	1.64 ± 0.40		1.15 ± 0.26		1.41 ± 0.43		0.85 ± 0.11	
*SNAP23*	1.00 ± 0.10	1.21 ± 0.11		0.91 ± 0.10		0.98 ± 0.11		0.93 ± 0.06	
*STX5*	1.00 ± 0.05	0.91 ± 0.06		0.87 ± 0.05		0.83 ± 0.03	*	0.94 ± 0.04	

Adipogenic Markers	RD+Veh	HFD+Veh	Sig.	HFD+0.3mg	Sig.	HFD+1.0mg	Sig.	HFD+5.0mg	Sig.
*ADRB3*	1.00 ± 0.09	1.45 ± 0.23		0.84 ± 0.04	°	1.47 ± 0.08	* ##	1.19 ± 0.07	## ^
*BMP4*	1.00 ± 0.14	1.31 ± 0.39		0.64 ± 0.05	*	0.73 ± 0.08		0.72 ± 0.13	
*BMP7*	1.00 ± 0.22	1.93 ± 0.33	*	0.40 ± 0.03	°	0.78 ± 0.15	°	0.37 ± 0.10	* °
*TMEM26*	1.00 ± 0.45	0.44 ± 0.13		1.35 ± 0.29	°	0.47 ± 0.17		1.66 ± 0.34	° ^

Inflammation Markers	RD+Veh	HFD+Veh	Sig.	HFD+0.3mg	Sig.	HFD+1.0mg	Sig.	HFD+5.0mg	Sig.
*COX2*	1.00 ± 0.29	3.04 ± 0.69	*	1.30 ± 0.65	°	1.77 ± 0.56		0.86 ± 0.14	°°
*GATA3*	1.00 ± 0.27	1.98 ± 0.21	*	1.03 ± 0.30		1.17 ± 0.13	°	1.12 ± 0.19	°
*IL-1β*	1.00 ± 0.39	2.00 ± 0.43		0.83 ± 0.33		0.74 ± 0.38		0.30 ± 0.03	°
*IL-6*	1.00 ± 0.34	1.53 ± 0.57		1.20 ± 0.55		0.78 ± 0.06		0.37 ± 0.07	° ^^
*IL-8*	1.00 ± 0.44	0.92 ± 0.47		0.49 ± 0.20		0.51 ± 0.19		0.05 ± 0.01	°° ## ^^
*TBX21*	1.00 ± 0.21	1.30 ± 0.26		0.76 ± 0.11		0.70 ± 0.08		0.57 ± 0.16	
*TLR2*	1.00 ± 0.25	2.16 ± 0.44		0.96 ± 0.09		1.05 ± 0.20		1.06 ± 0.03	
*TLR4*	1.00 ± 0.13	1.65 ± 0.25		0.75 ± 0.14	°	0.66 ± 0.15	°	0.79 ± 0.17	
*TNFα*	1.00 ± 0.15	2.07 ± 0.65		1.46 ± 0.52		0.97 ± 0.33		0.88 ± 0.22	

Results are expressed as fold change vs RD+Veh and reported as mean ± SEM (RD+Veh, n=6; HFD+Veh, n=6; HFD + 0.3mg, n=6; HFD + 1.0mg, n=6; HFD + 5.0mg, n=6). Statistical analysis (Sig.) was performed using one-way non-parametric ANOVA Kruskal-Wallis test followed by post hoc Mann-Whitney analysis.

*p<0.05 vs RD+Veh; °p<0.05 vs HFD+Veh; ^##^p<0.05 vs HFD + 0.3mg; ^p<0.05, ^^p<0.01 vs HFD + 1.0mg.

Notably, the mRNA expression of *GATA3* and *TBX21*, transcription factors involved in immune cell polarization, was reduced in all treatment groups, compared to HFD+Veh.

**Table 2** presents the mRNA expression levels in VAT homogenates for the same markers that were previously analyzed in DIM-differentiated adipocytes. Overall, the expression patterns observed in the tissue closely mirror those detected in the cultured cells. This strong concordance suggests that the *in vitro* adipocyte model reliably recapitulates the molecular profile of the directly harvested visceral adipose tissue.

**Table 2:** Biomarkers mRNA expression in VAT homogenates.

Mitochondrial Markers	RD+Veh	HFD+Veh	Sig.	HFD+0.3mg	Sig.	HFD+1.0mg	Sig.	HFD+5.0mg	Sig.
*FIS1*	1.00 ± 0.09	1.22 ± 0.12		0.98 ± 0.15		0.91 ± 0.13		0.98 ± 0.14	
*MFN1*	1.00 ± 0.12	1.08 ± 0.14		1.11 ± 0.15		0.99 ± 0.13		1.05 ± 0.10	
*MFN2*	1.00 ± 0.07	1.12 ± 0.09		0.66 ± 0.14	°	0.91 ± 0.09		0.80 ± 0.13	
*OPA1*	1.00 ± 0.13	1.20 ± 0.13		0.99 ± 0.15		0.85 ± 0.09	°	0.76 ± 0.11	°
*TFAM*	1.00 ± 0.10	1.36 ± 0.15		0.99 ± 0.16		1.03 ± 0.13		0.90 ± 0.07	°

LD storage Markers	RD+Veh	HFD+Veh	Sig.	HFD+0.3mg	Sig.	HFD+1.0mg	Sig.	HFD+5.0mg	Sig.
*PLIN1*	1.00 ± 0.20	1.51 ± 0.30		0.97 ± 0.18		0.95 ± 0.17		0.78 ± 0.14	
*SNAP23*	1.00 ± 0.13	1.34 ± 0.19		1.12 ± 0.17		0.92 ± 0.14		1.09 ± 0.13	
*STX5*	1.00 ± 0.10	0.99 ± 0.11		0.86 ± 0.13		0.79 ± 0.11		0.85 ± 0.09	

Adipogenic Markers	RD+Veh	HFD+Veh	Sig.	HFD+0.3mg	Sig.	HFD+1.0mg	Sig.	HFD+5.0mg	Sig.
*ADRB3*	1.00 ± 0.13	1.40 ± 0.28		0.93 ± 0.16		1.11 ± 0.16		0.96 ± 0.14	
*BMP4*	1.00 ± 0.26	1.35 ± 0.19		0.76 ± 0.23	°	0.83 ± 0.17		0.80 ± 0.18	
*BMP7*	1.00 ± 0.17	1.82 ± 0.56	*	0.89 ± 0.16	°	1.08 ± 0.19		0.74 ± 0.12	°°
*TMEM26*	1.00 ± 0.25	0.86 ± 0.16		1.10 ± 0.25		0.79 ± 0.25		0.92 ± 0.16	

Inflammation Markers	RD+Veh	HFD+Veh	Sig.	HFD+0.3mg	Sig.	HFD+1.0mg	Sig.	HFD+5.0mg	Sig.
*COX2*	1.00 ± 0.22	2.02 ± 0.25	*	1.26 ± 0.26	°	1.63 ± 0.45		1.31 ± 0.10	°
*GATA3*	1.00 ± 0.10	1.48 ± 0.22		1.06 ± 0.18		1.32 ± 0.22		0.98 ± 0.10	°
*IL-1β*	1.00 ± 0.22	1.73 ± 0.61		0.85 ± 0.28		0.64 ± 0.13		0.89 ± 0.29	
*IL-6*	1.00 ± 0.17	1.47 ± 0.38		1.07 ± 0.24		0.66 ± 0.19		0.73 ± 0.11	
*IL-8*	1.00 ± 0.45	1.01 ± 0.23		0.52 ± 0.17		0.52 ± 0.13		0.39 ± 0.07	
*TBX21*	1.00 ± 0.17	1.24 ± 0.37		0.98 ± 0.14		0.87 ± 0.29		0.86 ± 0.18	
*TLR2*	1.00 ± 0.16	1.61 ± 0.34		1.07 ± 0.25		0.80 ± 0.15	°	0.73 ± 0.14	°
*TLR4*	1.00 ± 0.14	1.67 ± 0.22	*	0.94 ± 0.16	°	0.87 ± 0.14	°	0.71 ± 0.12	°
*TNFα*	1.00 ± 0.18	1.61 ± 0.49		1.49 ± 0.76		0.94 ± 0.27		0.75 ± 0.10	

Results are expressed as fold change vs RD+Veh and reported as mean ± SEM (RD+Veh, n=6; HFD+Veh, n=7; HFD + 0.3mg, n=7; HFD + 1.0mg, n=7; HFD + 5.0mg, n=7). Statistical analysis (Sig.) was performed using one-way non-parametric ANOVA Kruskal-Wallis test followed by post hoc Mann-Whitney analysis.

*p<0.05 vs RD+Veh; °p<0.05, °°p<0.01 vs HFD+Veh.

Most likely, the lack of significance when analyzing several molecular markers is due to the relatively high intragroup variability accompanied to the small size of the groups. However, trends of mRNA expression still clearly show a beneficial effect of selvigaltin treatment regarding the HFD-induced biomarkers alterations.

## 4 Discussion

The present study is a spinoff of a previous larger study aimed at investigating the effect of a galactin-3 inhibitor (selvigaltin) on MASLD (Comeglio et al., 2024). During the course of that study, we noticed that selvigaltin induced a consistent decrease in HFD-induced VAT accumulation. The aim of the present study was to investigate in depth the metabolic phenotype of visceral preadipocytes isolated from a subset of rabbits treated *in vivo* with selvigaltin, in comparison with those obtained from untreated HFD animals and healthy controls. rPADs were collected at the end of the *in vivo* treatment, subsequently cultured and induced *in vitro* to become DIM-differentiated adipocytes.

This approach allowed us to assess the effects of *in vivo* pharmacological inhibition of Gal-3 on the functionality of DIM-differentiated adipocytes, focusing on key features of mature adipocyte biology, such as mitochondrial morphology and respiration, ATP production, adipogenic differentiation capacity, and inflammatory marker expression. Morphological, molecular, and functional analyses of DIM-differentiated visceral preadipocytes demonstrate that selvigaltin exerts a broad protective effect on mitochondrial structure, oxidative balance, and inflammatory signalling pathways.

All functional assessments were performed in DIM-differentiated adipocytes derived from isolated preadipocytes, confirming that the observed effects of *in vivo* selvigaltin administration accurately reflect adipocyte functionality under controlled experimental conditions. As previously reported, mitochondrial dysfunction is a defining feature of metabolically impaired adipocytes in MetS (Wang et al., 2013; Woo et al., 2019; Heinonen et al., 2020). Mitochondrial dysfunction is a central factor in visceral fat accumulation, as it disrupts lipid oxidation, elevates oxidative stress, and triggers chronic inflammation (Xia et al., 2024). In obese individuals with hypertriglyceridemia, visceral adipose tissue exhibits heightened mitophagy and reduced mitochondrial content, which aligns with impaired lipid metabolism (Mela et al., 2022).

This mitochondrial impairment can persist even after weight loss, reflecting sustained metabolic reprogramming (Breininger et al., 2019). Reactive oxygen species generated by dysfunctional mitochondria activate pro-inflammatory pathways within visceral fat depots (Parasiliti-Caprino et al., 2022).

In our model, HFD exposure induced substantial alterations in mitochondrial morphology, including reduced length, motility, and cristae area, hallmarks of fragmentation and compromised bioenergetic integrity (Jheng et al., 2012). *In vivo* selvigaltin restored these parameters largely in a dose-dependent fashion, with mitochondrial length and velocity normalized at doses ≥1.0 mg/kg. This suggests enhanced fusion-fission dynamics and organelle turnover, in line with previous reports linking mitochondrial dynamics to adipocyte metabolic flexibility (Liesa and Shirihai, 2013; Rovira-Llopis et al., 2017). Likewise, cristae area was significantly improved by treatment, reaching normalized values at low doses (0.3-1.0 mg/kg), while the cristae number showed a discernible trend toward recovery.

These benefits were accompanied by a marked attenuation of oxidative stress, with DHE fluorescence imaging revealing a strong reduction in ROS accumulation in *in vivo* selvigaltin-treated DIM-differentiated adipocytes. The correlation between ROS levels and mitochondrial length further supports a causal link between organelle integrity and redox homeostasis. These findings align with the view that mitochondrial fragmentation exacerbates oxidative stress in dysfunctional adipocytes (Furukawa et al., 2004; Mailloux et al., 2013; Maneschi et al., 2013; Comeglio et al., 2018). In addition, selvigaltin modulated lipid storage capacity, where HFD-induced LD hypertrophy and increased droplet number were reversed by *in vivo* selvigaltin treatment, with LD volume returning to values comparable to healthy controls at higher doses.

These changes were paralleled by the transcriptional downregulation of *PLIN1* and *SNAP23*, suggesting reduced lipid accumulation and membrane trafficking (Jägerström et al., 2009; Sztalryd and Brasaemle, 2017), and pointing to a functional reprogramming of DIM-differentiated adipocytes toward a metabolically favourable phenotype (Wu et al., 2012; Harms and Seale, 2013).

Notably, in DIM-induced adipocytes *BMP4* and *BMP7* mRNA expression was substantially downregulated by selvigaltin in a dose-dependent manner, compared to HFD+Veh and controls. Both variables showed a similar trend, although only BMP7 reached statistical significance. While both factors are implicated in adipogenic and browning programs (Wu et al., 2012; Modica and Wolfrum, 2013), some studies suggests that their overexpression in the context of obesity may contribute to maladaptive VAT remodelling and fibrosis (Tseng et al., 2008; Modica et al., 2016). Thus, the observed suppression of *BMP4* and *BMP7* may reflect a normalization of pathological adipogenic signalling, consistent with improved tissue homeostasis following Gal-3 inhibition (Pejnovic et al., 2013).

Inflammatory markers gene expression was also significantly attenuated in these cells. HFD induced a marked upregulation of canonical inflammatory markers (*COX2, IL-6*, *TLR2*, *TLR4*, *TNFα*), all of which were appreciably reduced across selvigaltin-treated groups. These effects likely reflect the known role of Gal-3 in modulating macrophage polarization and cytokine release within VAT (Sano et al., 2003; Pejnovic et al., 2013; Li et al., 2014; Martínez-Martínez et al., 2016). Considering *IL-1β*, *IL-8*, and transcription factors such as *GATA3* and *TBX21* evident changes, a general anti-inflammatory shift was clear in the mRNA expression profile of selvigaltin-treated groups.

The similarity between markers expression in VAT and DIM-differentiated adipocytes suggests that the behavior of the latter reflects the physiological state of the donor animal’s visceral adipose tissue. This concordance provides additional confidence that the *in vitro* differentiation results are representative of *in vivo* conditions and support the relevance of our findings to metabolic health in the different experimental groups.

While SDH activity showed a significant change in the HFD+Veh group, normalized by higher doses of selvigaltin, ATP production did not quite reach statistical significance. However, both parameters exhibited a consistent upward trend following selvigaltin administration. This is consistent with previous evidence showing that improving mitochondrial structure enhances oxidative phosphorylation efficiency and cellular bioenergetics (Pagliarini et al., 2008; Guerrier et al., 2023).

These results, taken together with the normalization of mitochondrial morphology and suppression of oxidative stress, following HFD-induced abnormalities, suggest a partial restoration of mitochondrial function that may require further exploration under more sensitive or prolonged experimental conditions.

Some limitations of the present study should be acknowledged. First, the sample size of experimental animals per group was relatively small, which may restrict the generalization of minor phenotypic trends. Nevertheless, it provides approximately 80% power to detect biologically meaningful effects, consistent with prior preclinical reports, also balancing the statistical rigor with the ethical principle of responsible animal use. Second, since mRNA levels do not always mirror functional protein abundance due to post-transcriptional modifications and varying translation efficiencies, our findings regarding inflammatory factors and adipocyte-specific markers should be interpreted as potential pathways under active regulation rather than definitive protein-level changes. Future studies utilizing high-throughput proteomics and functional mitochondrial respiration assays are warranted to fully validate these metabolic transitions at the translational level. Finally, a limitation of this study is that analyses were primarily performed on preadipocytes isolated from VAT, with *ex vivo* assessments focusing on differences in visceral fat between groups. While DIM-induced differentiation reflects the health status of the donor animal’s preadipocytes, this approach does not capture all *in vivo* complexities. mRNA analyses in VAT homogenates largely mirrored the findings in differentiated adipocytes, but results should still be interpreted with this limitation in mind.

Oral Gal-3 inhibitor selvigaltin has been shown to significantly reduce visceral fat in high-fat diet models, potentially by mitigating Gal-3-driven inflammation. In diet-induced obese mice, Gal-3 mRNA and protein are markedly enriched in the stromal-vascular fraction of VAT, with the highest intracellular levels detected in CD11c+ pro-inflammatory adipose tissue macrophages (Rhodes et al., 2013). Consistent with these data, human and mouse studies have shown that Gal-3 is expressed by both adipocytes and infiltrating macrophages (Pang et al., 2013; Baek et al., 2015). By modulating the inflammatory microenvironment, selvigaltin may support mitochondrial restoration and metabolic improvement. Overall, this study expands the current understanding of Gal-3 contribution to VAT dysfunction and identifies selvigaltin as a promising pharmacological tool to counteract metabolic-syndrome induced visceral adipocyte homeostasis and health alterations. By promoting mitochondrial remodelling, limiting oxidative stress, and dampening inflammation, Gal-3 inhibition may offer therapeutic benefits in metabolic disorders characterized by visceral obesity and adipose tissue dysfunction.

Additional research is needed to clarify its therapeutic value in treating visceral obesity.

## Data Availability

The original contributions presented in the study are included in the article/**Supplementary Material**. Further inquiries can be directed to the corresponding author.

## Glossary

ADRB3: beta-3 adrenergic receptor
BMP4: bone morphogenetic protein 4
BMP7: bone morphogenetic protein 7
BW: body weight
CD45: cluster of differentiation 45
COX2: inducible cyclooxygenase-2
DHE: dihydroethidium
DIM: differentiation induction medium
FIS1: mitochondrial fission 1 protein
FN1: fibronectin
Gal-3: galectin-3
GATA3: GATA binding protein 3
HFD: high-fat diet
IL-1β: interleukin-1, subunit beta
IL-6: interleukin-6
IL-8: interleukin-8
LD: lipid droplet
MASH: metabolic-associated steatohepatitis
MASLD: metabolic dysfunction-associated steatotic liver disease
MetS: metabolic syndrome
MFN1: mitofusin-1
MFN2: mitofusin-2
NAFLD: non-alcoholic fatty liver disease
NASH: non-alcoholic steatohepatitis
OPA1: dynamin-like 120 kDa protein, mitochondrial
PECAM1: platelet endothelial cell adhesion molecule
PLIN: lipid droplet-associated protein, perilipin 1
PPARγ: peroxisome proliferator-activated receptor gamma
RD: regular diet
ROI: region of interest
ROS: reactive oxygen species
rPADs: rabbit preadipocytes
SM22: transgelin
SNAP23: synaptosomal-associated protein 23
STX5: syntaxin-5
TBX21: T-box transcription factor TBX21
TEM: transmission electron microscopy
TFAM: mitochondrial transcription factor A
TLR2: toll-like receptor 2
TLR4: toll-like receptor 4
TMEM26: transmembrane protein 26
TNFα: tumor necrosis factor alpha
VAT: visceral adipose tissue.
