# Supplementary material for: Galectin-3 inhibitor selvigaltin dosed therapeutically in vivo in a rabbit model of metabolic syndrome induced by high-fat diet: effects on rabbit differentiated adipocytes
Source: Front Physiol. 2026 Jul 9;17:1710592. doi: 10.3389/fphys.2026.1710592 (PMC13391330; doi:10.3389/fphys.2026.1710592)
Supplement: Supplementary file 1 [file Table1.docx]

**Supplementary Table ST1.** Clinical and biochemical data at sacrifice.

| **Variable** | **RD+Veh** | **HFD+Veh** | **Sig.** | **HFD+0.3mg** | **Sig.** | **HFD+1.0mg** | **Sig.** | **HFD+5.0mg** | **Sig.** |
| --- | --- | --- | --- | --- | --- | --- | --- | --- | --- |
| **Glycaemia (gr/L)** | 1.21 ± 0.15 | 1.79 ± 0.08 | ***** | 1.41 ± 0.15 |  | 1.43 ± 0.07 | **°** | 1.38 ± 0.14 |  |
| **OGTT (iAUC)** | 139.72 ± 8.34 | 183.38 ± 10.75 | ****** | 165.30 ± 9.26 |  | 184.78 ± 8.41 | ***** | 165.05 ± 12.37 |  |
| **Plasma Cholesterol (mg/dL)** | 28.00 ± 6.52 | 2,656.67 ± 268.71 | ****** | 2,352.67 ± 474.02 | ****** | 2,286.67 ± 324.61 | ****** | 1,520.17 ± 153.62 | **** °** |
| **Plasma Triglycerides (mg/dL)** | 70.50 ± 5.85 | 139.83 ± 16.21 | ****** | 150.83 ± 21.82 | ***** | 134.50 ± 23.44 | ***** | 122.00 ± 21.76 | ***** |
| **MAP (mmHg)** | 97.60 ± 8.46 | 148.13 ± 9.97 | ***** | 145.73 ± 7.17 | ****** | 151.23 ± 10.70 | ****** | 141.04 ± 12.46 | ***** |
| **AST (U/L)** | 28.23 ± 4.31 | 84.33 ± 16.73 | ****** | 60.50 ± 16.26 |  | 55.67 ± 11.02 |  | 61.67 ± 7.01 | ***** |
| **ALT (U/L)** | 27.50 ± 1.96 | 73.50 ± 14.21 | ****** | 48.00 ± 9.70 |  | 57.83 ± 12.39 |  | 51.33 ± 6.62 | ***** |

*iAUC, incremental area under the curve of glucose blood level during oral glucose tolerance test (OGTT); MAP, mean arterial pressure; AST, aspartate aminotransferase; ALT, alanine aminotransferase. Data are expressed as mean±SEM (RD+Veh, n=6; HFD+Veh, n=6; HFD+0.3mg, n=6; HFD+1.0mg, n=6; HFD+5.0mg, n=6). To evaluate differences between groups, with p<0.05 considered as significant, statistical analysis (Sig.) was performed using one-way non-parametric Kruskal-Wallis ANOVA followed by post hoc Mann-Whitney analysis. * p<0.05, ** p<0.01 vs RD+Veh; ° p<0.05 vs HFD+Veh*

*The datasets analyzed in this study are subsets of the larger dataset previously described (41) and are presented here to confirm that the HFD group mostly consists of animals affected by metabolic syndrome.*
